# Insulin-self administration among individuals with diabetes: Implications for improved practices

**DOI:** 10.1371/journal.pone.0324846

**Published:** 2025-06-05

**Authors:** Anan S. Jarab, Walid A. Al-Qerem, Salam Alqudah, Karem H. Alzoubi, Shrouq R. Abu Heshmeh, Yazid N. Al Hamarneh, Eman Alefishat, Yousef Mimi, Maher Khdour

**Affiliations:** 1 College of Pharmacy, Al Ain University, Abu Dhabi, United Arab Emirates; 2 AAU Health and Biomedical Research Center, Al Ain University, Abu Dhabi, United Arab Emirates; 3 Department of Pharmacy, Faculty of Pharmacy, Al-Zaytoonah University of Jordan, Amman, Jordan; 4 Department of Pharmacy, Jordanian Royal Medical Services, Amman, Jordan; 5 Department of Pharmacy Practice and Pharmacotherapeutics, University of Sharjah, Sharjah, United Arab Emirates; 6 Faculty of Pharmacy, Jordan University of Science and Technology, Irbid, Jordan; 7 Department of Pharmacology, Faculty of Medicine and Dentistry, University of Alberta, Edmonton, Canada; 8 Department of Medical Sciences, College of Medicine and Health Science, Khalifa University of Science and Technology, Abu Dhabi, United Arab Emirates; 9 Department Biopharmaceutics and Clinical Pharmacy, Faculty of Pharmacy, The University of Jordan, Amman, Jordan; 10 Center for Biotechnology, Khalifa University of Science and Technology, Abu Dhabi, United Arab Emirates; 11 Department of Health Sciences, Faculty of Graduate Studies, Arab American University, Jenin, Palestine; 12 College of Pharmacy, Al-Quds University, Jerusalem, Palestine; UCSI University, MALAYSIA

## Abstract

**Background:**

Diabetes significantly contributes to both microvascular and macrovascular complications. Effective management depends on meticulous glycemic control, with insulin playing a crucial role. The success of insulin therapy relies on patients’ ability to properly administer insulin and adhere to the administration instructions.

**Objective:**

This cross-sectional study aimed to evaluate the knowledge and practices of insulin use among patients with type 1 and type 2 diabetes and to identify factors influencing these practices.

**Methods:**

A validated, self-administered questionnaire was distributed in person to outpatient insulin users with type 1 and type 2 diabetes at King Abdullah University Hospital. In addition to socio-demographics and health characteristics, the questionnaire evaluated patients’ knowledge regarding insulin, its administration, and insulin use practices. Quantile regression was used to explore factors associated with insulin administration practices.

**Results:**

The study included 402 patients, 53.0% of which are females, with a median age of 54 years. The median (interquartile range) knowledge score was 5 (4–6) out of a maximum possible score of 9, while the median (interquartile range) insulin administration practice score was 80.39 (72.92–85.42) out of a maximum possible score of 100. = . Lower practice levels were associated with older age (coefficient: −0.149, 95%CI: −0.217- −0.082), lack of diabetes information (coefficient: −6.189, 95%CI: −12.041 - −0.337), and reliance on non-scientific information sources (coefficient: −2.409, 95%CI: −4.562 - −0.255). However, higher knowledge scores were associated with better practices (coefficient: 2.516, 95%CI: 1.819–3.213).

**Conclusions:**

While the study reveals acceptable knowledge and practices regarding insulin self-administration, it also highlights significant gaps that policy initiatives should address by implementing uniform training programs and interventions to promote effective insulin administration practices.

## Introduction

Diabetes mellitus is a chronic metabolic disorder characterized by impaired insulin secretion or insulin resistance, resulting in elevated blood glucose levels. This dysfunction often arises from pancreatic beta-cell dysfunction or insufficient insulin production [1].

Insufficient insulin production characterizes type 1 diabetes, necessitating daily insulin administration, while insulin resistance primarily underlies type 2 diabetes [1]. According to the World Health Organization, approximately 422 million individuals globally suffer from diabetes, with the majority residing in low- and middle-income countries. The disease is directly attributed to 1.5 million deaths annually, with a steady rise in the prevalence of diabetes over recent decades [2].

Diabetes is a leading cause of stroke, heart attacks, kidney failure, blindness, and lower limb amputations. The age-standardized death rate from diabetes increased by 3% between 2000 and 2019 [1]. Maintaining blood sugar levels within normal ranges is the primary goal of diabetes management.

Insulin is an essential medication for regulating blood glucose levels, particularly in the management of individuals with type 1 diabetes. Furthermore, it frequently supplements oral hypoglycemic agents in type 2 diabetes patients who have not achieved their glycemic control targets [3]. Despite its demonstrated effectiveness, the effective utilization of insulin therapy depends not only on the availability and accessibility of insulin but also on patients’ knowledge and practices regarding self-administration of insulin. For patients to effectively manage their condition, it is imperative that they have knowledge about insulin therapy, including administration techniques and potential side effects [4]. Previous research found a significant association between good knowledge and good insulin use practices [4–7]. Other studies reported several factors associated with insulin-self-administration practice, including gender, education, occupation, duration of insulin therapy, and duration of diabetes [7,8]. The aim of the current study was to evaluate knowledge and practices regarding insulin use among people with type 1 and type 2 diabetes in Jordan, as well as the factors associated with insulin use practice. This study acknowledges the existing literature on insulin self-administration but, to the best of our knowledge, provides the first investigation within the context of the Middle East. It provides unique insights by specifically addressing gaps in users’ knowledge and practices in a region where diabetes is increasingly prevalent. Unlike most previous studies, the present research targets both individuals with type 1 diabetes and insulin users among patients with type 2 diabetes. This approach provides valuable insights into insulin administration practices and enhances the understanding of how insulin management is approached by diverse patient groups, potentially informing more effective support strategies for individuals with diabetes. Given the discrepancies in the factors identified in the literature, the findings of this study further narrow down the key factors associated with poor insulin-administration practices, which is crucial for informing tailored interventions that can improve self-management and ultimately enhance health outcomes among insulin users in both type 1 and type 2 diabetes populations.

## Methods

### Study design and subjects

The current cross-sectional study was conducted on insulin users with type 1 and type 2 diabetes attending the outpatient diabetes clinic at King Abdullah University Hospital (KAUH) from February to August 2022, using a convenient sampling technique. Patients who had chronic follow-up visits at KAUH during the study period were eligible to be included in the study. Patients under 18 years of age, those with type 2 diabetes who were not using insulin therapy, severely ill patients experiencing short-term health conditions, such as infections or diabetic ketoacidosis, that could interfere with their ability to participate or accurately report insulin self-administration, as well as those attending for diabetic complications, unable to communicate, or who required assistance with insulin administration, were excluded from the study. The researcher approached patients during their outpatient clinic visits, explained the study’s objectives, and emphasized the voluntary nature of participation and the confidentiality and anonymity of the study findings. It took an average of 10–15 minutes to complete the questionnaire.

### Ethical approval statement

The study received ethical approval by the Institutional Review Board (IRB) of King Abdullah University Hospital (Ref. # 24/138/2021). Written informed consent was obtained from all participants before they completed the study questionnaire.

### Sample size calculation

Green’s rule, which states that “n = 50 + 8*predictors,” was utilized to calculate the required sample size [9]. The largest model in this study included eight predictors. Therefore, according to Green’s rule, the minimum required sample size for this study is 114.

### Study instrument

After reviewing the relevant literature [10,11], the questionnaire for the current study was developed (S1 File). The first section collected sociodemographic information, including age, gender, marital status, education, type of diabetes, diabetes onset history, and family history of diabetes. These sociodemographic data were selected based on their relevance to understanding factors that could influence insulin self-administration practices, as well as their alignment with the existing literature on insulin self-administration and diabetes management and the specific objectives of this study. The second section included nine items assessing patients’ knowledge of insulin and its administration, using true/false responses for each item. Each correct response was assigned a score of one, while incorrect responses received a score of zero. The scores were summed, yielding a total score ranging from 0 to 9. The last section consisted of 20 items to evaluate insulin use practices, including 13 common items for both syringe and pen users, 4 additional questions for syringe users, and 3 for pen users. The participants were asked to answer the questions in this domain on a three-point Likert scale ranging from never (score = 1) to always (score = 3). The scores were calculated by summing the points obtained for each item. Given that the number of items differed among syringe users, pen users, and patients utilizing both devices, the scores were standardized by dividing the total score by the number of items and subsequently multiplying the result by 100. For patients who use both pen and syringe the average of the two scores was recorded. This transformation ensured a unified maximum possible score of 100 across all groups.

### Tool validation

The questionnaire was initially developed in English and translated into Arabic by a professional translator. It was then back translated into English by a second translator, and the two English versions were compared to ensure consistency and accuracy. This forward–backward translation process helped ensure that the Arabic version was both linguistically and culturally appropriate for the study population. The content validity of the questionnaire was evaluated by a panel of experts, including two endocrinologists and two clinical pharmacists. The experts confirmed that the questionnaire was comprehensive, encompassing the essential steps of insulin administration and key knowledge points while employing simplified and easily understandable terminology. To assess face validity, a pilot study was conducted with 30 randomly selected patients. Research assistants explained the study’s objectives and obtained informed consent. Participants then completed the questionnaire and provided feedback. An open discussion was facilitated to gather their opinions on the relevance of the items, ease of response, and clarity of the questions. The feedback was reviewed by the researchers and experts, resulting in minor modifications, including the substitution of synonyms to improve clarity and comprehension. Data from the pilot study was not included in the final analysis. Additionally, the internal consistency of the knowledge and of insulin use practice scales was assessed by computing Cronbach’s alpha, with an acceptable value of > 0.5, as lower Cronbach’s alpha values are acceptable for dichotomous data [12]. However, due to the variation in the number of items for each group (pen, syringe, or both users), three separate Cronbach’s alpha values were computed.

### Statistical analysis

The data analysis was performed using SPSS version 26. Categorical variables were presented as percentages and frequencies. The Kolmogorov Smirnov test revealed a lack of normality in the continuous variables, including insulin the administration practice score. Therefore, continuous variables were presented as medians and interquartile range (IQR), and quantile regression was used to explore the variables associated with the insulin administration practice score. The regression model included age, gender, marital and educational status, type of diabetes, disease duration, family history of diabetes, receipt of information about diabetes and whether the information came from scientific sources, and knowledge score as predictors, with the insulin administration practice score as the dependent variable (S.2). Significance was set at a threshold of p < 0.05.

## Results

The present study enrolled 402 patients, 53.0% of which were females, with a median (IQR) age of 54 (30–62) years. The majority of the patients were married (69.4%), had type 2 diabetes (62.9%), had diabetes for 10 years or more (52.0%), and had a family history of diabetes (63.9%). The sociodemographic characteristics of the participants are presented in Table 1.

**Table 1:** Sociodemographic and disease-related characteristics of the study participants (n = 402).

Variable		Frequency (%) or median (interquartile range)
Age (years)		54 (32)
Gender	Female	213 (53.0%)
	Male	189 (47.0%)
Marital status	other	123 (30.6%)
	married	279 (69.4%)
Educational level	Elementary	122 (30.3%)
	High school	103 (25.6%)
	College	57 (14.2%)
	University	120 (29.9%)
Type of diabetes	Type one	149 (37.1%)
	Type two	253 (62.9%)
Onset history of diabetes	1-4 years	93 (23.1%)
	5-9 years	100 (24.9%)
	10 years or more	209 (52.0%)
Family history of Diabetes	No	145 (36.1%)
	Yes	257 (63.9%)

The median (IQR) for the “knowledge towards the use of insulin” score was 5 (4-6) out of a maximum possible score of 9. As shown in Table 2, more than 90% of the participants correctly identified that insulin is a hormone used to reduce blood glucose levels (95%) and that the sites for insulin injection are the abdomen, upper arm, and thigh (91.3%). However, the lowest correct responses were for the statements” Long-acting insulin (Lantus/Basaglar/Toujeo, Levemir, Tresiba) could be injected at any time of the day regardless of the meal?” (50.5%) and “An insulin vial is stored in the refrigerator, but at first use, it could be stored at room temperature” (58.2%). The Cronbach’s alpha for the knowledge scale was 0.51.

**Table 2:** Knowledge about insulin use (n = 402).

Knowledge items	Incorrect answer	Correct answer
Insulin is a hormone used to reduce the level of glucose in the blood.	20 (5%)	382 (95%)
The most appropriate time to inject short-acting insulin (Novolin R, Velosulin) is after meals	118 (29.4%)	284 (70.6%)
Long-acting insulin (Lantus/Basaglar/Toujeo, Levemir, Tresiba) could be injected at any time of the day regardless of the meal	199 (49.5%)	203 (50.5%)
The sites for insulin injection are the abdomen, upper arm, and thigh	35 (8.7%)	367 (91.3%)
An insulin vial is stored in the refrigerator, but at first use, it could be stored at room temperature	168 (41.8%)	234 (58.2%)
Rolling the vial between your hands is necessary to warm the insulin and reduces pain	136 (33.8%)	266 (66.2%)
The angle of injection is 90 degrees for obese patients and 45 degrees for lean patients	126 (31.3%)	276 (68.7%)
The complications of insulin therapy are low blood sugar, insulin allergy, and lipodystrophy	89 (22.1%)	313 (77.9%)
The benefits of insulin self-administration are that it is time-saving, cheap, and easily portable.	108 (26.9%)	294 (73.1%)

The total median (IQR) practice score for pen and syringe users was 80.39 (72.92–85.42) out of a maximum possible score of 100. As shown in Table 3, only 16.9% of syringe users always wiped the top of the vial with 70% alcohol, while 25.6% of participants always cleaned the skin with alcohol and allowed it to dry before injecting insulin with either a pen or syringe. Regarding common practices, less than half of the participants always compressed the skin without massaging it (31.6%), checked glucose levels before and after insulin injection (37.3%), left the skin relaxed before administration (38.8%), did not shake the insulin vial/pen (44.3%), and always washed their hands (47.5%). Cronbach’s alpha for the practice scale was 0.72 for the syringe group, 0.70 for the pen group, and 0.78 for participants using both syringe and pen.

**Table 3:** Insulin administration practices among the study participants (n = 202).

Insulin administration Practices	Never	Sometimes	Always
Hand washing	64 (15.9%)	147 (36.6%)	191 (47.5%)
Checking my glucose level before and after insulin injection	58 (14.4%)	194 (48.3%)	150 (37.3%)
Observing the insulin characteristics (cloudiness, color, and presence of precipitation)	65 (16.2%)	63 (15.7%)	274 (68.2%)
Rolling insulin vial/pen between my hands	91 (22.6%)	84 (20.9%)	227 (56.5%)
Don’t shake the insulin vial/pen	161 (40%)	63 (15.7%)	178 (44.3%)
Checking if the insulin device is broken or damaged.	27 (6.7%)	58 (14.4%)	317 (78.9%)
I clean the skin with alcohol and allow it to dry	170 (42.3%)	129 (32.1%)	103 (25.6%)
I recap the needle up to the moment of administration	8 (2%)	5 (1.2%)	389 (96.8%)
I pinch a fold of skin	81 (20.1%)	84 (20.9%)	237 (59.0%)
I inject the needle at 45 or 90 degrees	22 (5.5%)	43 (10.7%)	337 (83.8%)
I leave the skin to be relaxed before administration	149 (37.1%)	97 (24.1%)	156 (38.8%)
I wait 5 seconds, then I withdraw the needle.	66 (16.4%)	50 (12.4%)	286 (71.1%)
I compress the skin without massaging it	158 (39.3%)	117 (29.1%)	127 (31.6%)
**Syringe users**			
Syringe users “I wipe the upper top of the vial with 70% alcohol”	72 (48.6%)	51 (34.5%)	25 (16.9%)
Syringe users “ I inject air in the insulin vial”	31 (20.9%)	19 (12.8%)	98 (66.2%)
Syringe users “I draw up the quantity of insulin necessary to complete the prescribed dose”	6 (4.1%)	34 (23%)	108 (73%)
Syringe users “I remove the bubbles from the syringe”	7 (4.8%)	16 (10.9%)	124 (84.4%)
**Pen users**			
Pen users “I wipe the upper top of the vial with 70% alcohol”	55 (17.7%)	52 (16.8%)	203 (65.5%)
Pen users “I set up the dial at zero after removing air from the needle”	27 (8.7%)	38 (12.3%)	245 (79%)
Pen users “I fix the prescribed dose of insulin”	11 (3.6%)	80 (26%)	217 (70.4%)

Most patients (90.8%) reported receiving information about diabetes. Of these, 72.1% relied on scientific resources and medical staff, while 27.9% relied at least partially on non-scientific sources, including social media and friends.

As shown in Table 4, the quantile regression results revealed that each unit increase in age significantly reduced the likelihood of having high insulin use practice (coefficient: −0.149, 95%CI: −0.217,-0.082). Conversely, each unit increase in knowledge score significantly increased the likelihood of high insulin use practice (coefficient: 2.516, 95%CI: 1.819–3.213). Patients who had not received information about diabetes had significantly lower practice scores compared to those who had (Coefficient: −6.189, 95%CI: −12.041, −0.337). Additionally, patients who relied at least partially on non-scientific sources for information had significantly lower practice scores when compared to those who relied solely on scientific sources (coefficient: −2.409, 95%CI: −4.562,-0.255).

**Table 4:** Factors associated with insulin use practices.

Parameter	Coefficient	p-value	95% Confidence Interval	
			Lower Bound	Upper Bound
(Intercept)	76.654	<0.001	70.822	82.487
Age	−0.149	<0.001	−0.217	−0.082
Knowledge score	2.516	<0.001	1.819	3.213
Gender				
Female	0.521	0.612	−1.499	2.541
Male	0	.	.	.
Marital status				
other	−0.927	0.503	−3.648	1.794
Married	0	.	.	.
Diabetes duration				
1-4 years	0.322	0.813	−2.353	2.997
5-9 years	1.206	0.328	−1.217	3.63
10 or more years	0	.	.	.
Education				
Elementary	−1.616	0.235	−4.285	1.053
High school	−1.965	0.174	−4.8	0.871
Community college degree	−1.995	0.225	−5.224	1.234
University degree	0	.	.	.
Patients have received information about DM				
No	−6.189	0.038	−12.041	−0.337
Yes	0	.	.	.
Source of information				
Non-Scientific sources	−2.409	0.028	−4.562	−0.255
Scientific resource	0	.	.	.

## Discussion

Proper insulin administration is a cornerstone of effective diabetes management, and errors in technique or understanding can compromise glycemic control and long-term health outcomes. This study contributes to the growing body of literature by evaluating knowledge and practices related to insulin use among insulin-treated diabetic patients in Jordan, a population where such investigations remain limited. While the overall practices were generally acceptable, our findings highlight key areas for improvement. Notably, age, knowledge level, prior receipt of diabetes-related information, and the sources of that information were significantly associated with insulin administration practices. These factors are particularly important given their potential link to treatment adherence and the risk of poor metabolic control, as supported by previous studies both within and outside the region. The moderate but insufficient level of knowledge observed in the present study is consistent with earlier studies [4,13,14], and higher than that reported in other studies [5,15–18]. However, the participants the present study also identified several knowledge gaps. Specifically, nearly half of the participants (49.5%) failed to recognize that long-acting insulin formulations could be injected at any time, regardless of meals, and 41.8% incorrectly answered the question regarding the storage conditions of insulin vials. Previous studies conducted in Ethiopia reported that one-third of the participants were unaware that insulin vials should be stored in the refrigerator at 2–8 °C [7,18]. These results suggest that while there is generally good knowledge about insulin use, certain areas still require improvement. Notably, there is a lack of knowledge regarding when to inject long-acting insulin and how to properly store insulin vials. Consequently, to maintain the effectiveness of insulin, healthcare professionals should prioritize patient education, providing clear and understandable instructions on when and how to inject different types of insulin, as well as proper storage conditions [19]. This ensures that patients are equipped with the necessary knowledge to administer their insulin therapy correctly and minimize potential complications.

Participants in the current study exhibited good to very good insulin administration practices, with room for improvement. Comparable findings were reported in a study conducted at the Dubai Diabetes Center, where the median percentage score was77.7% [20]. However, the latter study focused solely on type 2 diabetes patients. Fego et al. reported that 60% of diabetic insulin users attending a hospital in southwest of Ethiopia administered insulin injections incorrectly [14]. Another interview-based cross-sectional study found that only 55.4% of diabetic patients treated with insulin administered the drug correctly [7]. The variation in insulin administration practices across different settings can be attributed to differences in research instruments, populations studied, cultural contexts, and study environments. These disparities highlight the need for standardized patient education and accessible tools, such as visual aids and digital reminders, to mitigate errors across various settings. Although practice levels were generally acceptable, participants in the present study demonstrated poor practices in several aspects of insulin administration. Similar results were reported in previous studies [20–22], while other studies indicated better practice levels [4,14,16]. However, even those studies that reported better practices revealed areas for improvement. It is crucial to examine the key factors influencing patients’ insulin usage practices. In this study, older age was significantly associated with reduced insulin use practices. Older adults may face challenges in following their insulin regimens due to age-related issues such as reduced vision, decreased mobility, decreased dexterity, poor cognitive function, and dependence on others [23,24]. Therefore, there is a need to develop tailored diabetes management programs specifically for insulin users in this demographic. Furthermore, the current study found that higher knowledge about insulin use, receiving information about diabetes, and relying on scientific sources for diabetes-related information were significantly associated with better insulin usage practices. A positive relationship between knowledge and practice of insulin use has been observed in earlier research [4–7]. These findings suggest that to improve insulin use knowledge, educational programs and materials focusing on current, accurate information about insulin and its administration should be developed. Additionally, individuals with diabetes should be encouraged to rely solely on scientific sources for diabetes-related information and seek guidance from healthcare professionals, as only 64.4% of the current study participants consider medical staff their primary source of information. By improving knowledge and ensuring access to reliable diabetes-related information, insulin usage practices can be improved, leading to better health outcomes for diabetic patients.

### Study limitations

This study’s single-center and cross-sectional design limits the ability to explore the underlying reasons for variations in insulin self-administration practices and the generalizability of findings to broader populations. Future multi-center studies with longitudinal follow-ups are needed to validate these observations across diverse geographical and socio-economic contexts and evaluate the effectiveness of interventions aimed at improving practices. Additionally, the risk of social desirability bias, where participants may provide false positive responses to please the interviewer, and the exclusion of individuals with visual/reading impairments, may limit the generalizability of the findings from the self-administered questionnaire. Future studies utilizing community-based participatory approaches, such as verbal interviews and caregiver-assisted surveys, could help capture a wider range of perspectives and mitigate these limitations. Lastly, while this study included individuals without diabetes complications to better understand the core factors influencing insulin self-management and minimize the potential impact of complications, future research could benefit from including individuals with complications to explore how these factors may further affect insulin administration.

## Conclusion

Despite the acceptable level of knowledge and practices regarding insulin self-administration, the study highlights critical gaps that require attention in both local and global diabetes care frameworks. The insights gained are expected to inform the development of future interventions aimed at improving insulin self-administration practices, ultimately leading to better blood glucose control and health outcomes among insulin users. While the findings are specific to a particular institutional context, the global variation in insulin administration practices suggests that standardized patient education could enhance practices across different settings. It is crucial for policy initiatives to standardize patient training programs and promote collaborative research to identify scalable solutions tailored to diverse populations.

## Supporting information

S1 File Study questionnaire. (DOC)

S2 File The survey dataset for participants’ responses. (SAV)
