# Correction: Insulin-self administration among individuals with diabetes: Implications for improved practices

**DOI:** 10.1371/journal.pone.0341749

**Published:** 2026-01-27

**Authors:** Anan S. Jarab, Walid A. Al-Qerem, Salam Alqudah, Karem H. Alzoubi, Shrouq R. Abu Heshmeh, Yazid N. Al Hamarneh, Eman Alefishat, Yousef Mimi, Maher Khdour

There is an error in affiliation 1 for author Anan S. Jarab. The correct affiliation 1 is: Department of Clinical Pharmacy, Faculty of Pharmacy, Jordan University of Science and Technology. Irbid 22110, Jordan.
