# Supplementary material for: Insulin-self administration among individuals with diabetes: Implications for improved practices
Source: PLoS One. 2025 Jun 5;20(6):e0324846. doi: 10.1371/journal.pone.0324846 (PMC12140230; doi:10.1371/journal.pone.0324846)
Supplement: S1 File — (DOC) [file pone.0324846.s001.doc]

**Insulin-self administration among individuals with diabetes: Implications for improved practices**

**Dear Sir/Madam**

**We are conducting a survey that aims to identify knowledge gaps and areas for improvement in insulin self-administration practices among patients with type 1 and type 2 who are using insulin therapy.**

**Your individual privacy will be maintained in all published and written data resulting from the study. Participation is voluntary, and you have the right to withdraw at any time without any consequences.**

**The questionnaire will take approximately 5 to 10 minutes to complete.**

**Your valuable insights are essential to this study, and we truly appreciate your participation.**

**Thank you for your time and support.**

**I have read instructions and I agree to participate in this study:**

** Yes**

### Part 1:  Sociodemographic

1. Age: __________

2. Gender:

- Male
- Female

3. Marital status

- Other
- Married

4. Educational status

- Elementary
- Higher School
- College
- University

7. Type of Diabetes:

- Type 1
- Type 2

6. Onset history of Diabetes

- 1-4 years
- 5-9 years
- 10 years or more

9. Family history of Diabetes

- Yes
- No

**Part2: Knowledge regarding ISA among diabetic patients:**

**Kindly answer the following true/false questions:**

1. Insulin is a hormone used to reduce the level of glucose in the blood

- True
- False

2. The most appropriate time to inject short-acting insulin (Novolin R, Velosulin) is after meals

- True
- False

3. Long-acting insulin (Lantus/Basaglar/Toujeo, Levemir,Tresiba) could be injected at any time of the day regardless of the meal

- True
- False

4. The sites for insulin injection are the abdomen, upper arm and thigh

- True
- False

5. An insulin vial is stored in the refrigerator, but after the first use it could be stored at room temperature.

- True
- False

6. Rolling the vial between your hands is necessary to warm the insulin and reduces pain

- True
- False

7. The angel of injection is 90 degree for obese patients and 45 degree for lean patients

- True
- False

8. The complications of insulin therapy are low blood sugar, insulin allergy, and lipodystrophy

- True
- False

9. The benefits of insulin self-administration are that it is time-saving, cheap, and easily portable

- True
- False

**Part3: practice regarding ISA among diabetic patients:**

Kindly select how well you do the following actions when you take your insulin dose:

1. Hand washing

- Always
- Sometimes
- Never

1. Check my glucose level before and after insulin injection

- Always
- Sometimes
- Never

1. Observe the insulin characteristics (cloudiness, color and presence of precipitation)

- Always
- Sometimes
- Never

1. Rolling insulin vial/pen between my hands

- Always
- Sometimes
- Never

1. Do not shake the insulin vial/pen

- Always
- Sometimes
- Never

1. Checking if the insulin device is broken or damaged

- Always
- Sometimes
- Never

1. I clean the skin with alcohol and allow it to dry

- Always
- Sometimes
- Never

1. I recap the needle up to the moment of administration

- Always
- Sometimes
- Never

1. I pinch a fold of skin

- Always
- Sometimes
- Never

1. I inject the needle in 45 or 90 degrees

- Always
- Sometimes
- Never

1. I leave the skin to be relaxed before administration

- Always
- Sometimes
- Never

1. I wait 5 seconds, then I withdraw the needle

- Always
- Sometimes
- Never

1. I compress the skin without massaging it

- Always
- Sometimes
- Never

1. **For whom using insulin syringe, please answer the following questions:**

I wipe the upper top of the vial with 70% alcohol

- Always
- Sometimes
- Never

I inject air in the insulin vial

- Always
- Sometimes
- Never

I draw up the quantity of insulin necessary to complete the prescribed dose

- Always
- Sometimes
- Never

I remove the bubbles from the syringe

- Always
- Sometimes
- Never

1. **For whom using pen injector, please answer the following questions:**

I wipe the upper top of the vial with 70% alcohol

 Always

 Sometimes

 Never

I set up the dial at zero after removing air from the needle

 Always

 Sometimes

 Never

I fix the prescribed dose of insulin

 Always
  Sometimes

 Never

**الإعطاء الذاتي للأنسولين بين الأفراد المصابين بالسكري: تداعيات لتحسين الممارسات**

**عزيزي السيد / السيدة،**

**نحن نجري استبيانًا يهدف إلى تحديد الفجوات المعرفية والمجالات التي تحتاج إلى تحسين في ممارسات الإعطاء الذاتي للأنسولين بين المرضى المصابين بالسكري من النوع الأول والنوع الثاني الذين يستخدمون العلاج بالأنسولين.**

**سيتم الحفاظ على خصوصيتك الفردية في جميع البيانات المنشورة والمكتوبة الناتجة عن الدراسة. المشاركة طوعية، ولديك الحق في الانسحاب في أي وقت دون أي تبعات.**

**سيستغرق إكمال الاستبيان حوالي 5 إلى 10 دقائق.**

**تعد ملاحظاتك القيمة أساسية لهذه الدراسة، ونحن نقدر مشاركتك حقًا. شكرًا لوقتك ودعمك.**

**لقد قرأت التعليمات وأوافق على المشاركة في هذه الدراسة:**

** نعم**

**القسم الاول: معلومات عامه:**

1. العمر: ______
2. الجنس:

- ذكر
- انتى

1. الحالة الاجتماعية:

- أخرى
- متزوج/متزوجة

1. المستوى الدراسي:

- ابتدائي
- ثانوي
- كلية
- جامعة

ما نوع مرض السكري؟

- النوع الاول
- النوع الثاني

1. منذ متى تم تشخيصك بمرض السكري؟

- من 1 – 4 سنوات
- من 5 – 9 سنوات
- 10 سنوات او اكثر

1. هل يوجد احد من افراد عائلتك مصاب بمرض السكري؟

- نعم
- لا

**القسم الثاني: مدى معرفة مرضى السكري بعمليات حقن الانسولين:**

**يرجى الإجابة على الأسئلة التالية بنعم او لا:**

1. الأنسولين هو هرمون يُستخدم لخفض مستوى السكر في الدم

- نعم
- لا

1. الوقت المناسب لحقن الانسولين قصير المدى هو بعد تناول وجبة الطعام مباشره

- نعم
- لا

1. يمكن حقن الانسولين طول المدى في اي وقت خلال اليوم بغض النظر عن الطعام

- نعم
- لا

1. مناطق حقن الانسولين في الجسم هي: البطن، اعلى الذراع، الفخذ

- نعم
- لا

1. يتم تخزين عبوة الانسولين في الثلاجة، ولكن بعد أول استخدام يمكن تخزينها في درجة حرارة الغرفة:

- نعم
- لا

1. تدوير عبوة الأنسولين بين راحتي اليد يساعد على تدفئة الأنسولين وتقليل الألم:

- نعم
- لا

1. زاوية الحقن هي 90 درجة للمرضى الذين يعانون من السمنة و45 درجة للمرضى النحيفين

- نعم
- لا

1. تتمثل مضاعفات العلاج بالأنسولين في انخفاض نسبة السكر في الدم ، وحساسية الأنسولين ، وهزال الأنسجة تحت الجلد

- نعم
- لا

9. من فوائد الحقن الذاتي للأنسولين أنه يوفر الوقت، غير مكلف، وسهلة الحمل

- نعم
- لا

**القسم الثالث: مدى تطبيق مرضى السكري لحق الانسولين:**

**يرجى اختيار مدى قيامك بالخطوات التالية عند اخذك لجرعة الانسولين:**

1. غسل اليدين

- دائما
- احيانا
- ابدا

1. فحص مستوى السكر بالدم قبل وبعد حقنة الانسولين

- دائما
- احيانا
- ابدا

1. اتفحّص الانسولين قبل اخذ الجرعة (أتأكد من لونه وعكورته ووجود الرواسب)

- دائما
- احيانا
- ابدا

1. تدوير عبوة الانسولين أو القلم بين راحتي يدي قبل حقنها

- دائما
- احيانا
- ابدا

1. تجنّب رج عبوة الأنسولين أو القلم دائما

- احيانا
- ابدا

1. التأكد من سلامة جهاز الأنسولين وعدم وجود كسر أو عطل قبل الاستخدام

- دائما
- احيانا
- ابدا

1. اقوم بتعقيم الجلد باستخدام الكحول واتركه حنى يجف

- دائما
- احيانا
- ابدا

1. اقوم بتغطية الإبرة حتى لحظة الحقن

- دائما
- احيانا

 ابدا

1. اقوم بقرص الجلد قبل حقن الإبرة

- دائما
- احيانا
- ابدا

1. اقوم بحقن الإبرة بزاوية 45 او 90 درجه

- دائما
- احيانا
- ابدا

1. اترك الجلد مسترخيًا ومن ثم احقن الجرعة المطلوبة

- دائما
- احيانا
- ابدا

1. انتظر 5 ثواني ومن ثم اقوم بإخراج الإبرة

- دائما
- احيانا
- ابدا

1. اضغط على مكان الحقنه دون تدليكها

- دائما
- احيانا
- ابدا

1. **ان كنت من مستخدمي الابر لحقن الانسولين اجب على الاسئلة الأربعة التالية:**

• امسح أعلى عبوة الأنسولين بالكحول 70 %

 دائما

 احيانا

 ابدا

• اقوم بسحب الهواء وحقنه بعبوة الانسولين

 دائما

 احيانا

 ابدا

• اسحب الكمية المطلوبة من الانسولين حسب الجرعة التي وصفها لي الطبيب

 دائما

 احيانا

 ابدا

• اقوم بإزالة فقاعات الهواء من الابرة قبل الحقن

 دائما

 احيانا

 ابدا

1. **ان كنت من مستخدمي القلم لحقن الانسولين اجب على الاسئلة الثلاث التالية**

• امسح أعلى عبوة الأنسولين بالكحول 70%

 دائما

 احيانا

 ابدا

• اقوم بضبط العجل الدوار على رقم صفر بعد إزالة الهواء من الإبرة

 دائما

 احيانا

 ابدا

• اقوم بضبط الجرعة المطلوبة التي وصفها لي الطبيب

 دائما

 احيانا

 ابدا
